# Causes and effects of theory-practice gap during clinical practice: the lived experiences of baccalaureate nursing students

**DOI:** 10.1080/17482631.2023.2164949

**Published:** 2023-01-19

**Authors:** Stella Ngozika Ugwu, Ngozi P. Ogbonnaya, Veronica Chiegeiro Chijioke, Judith Njideka Esievo

**Affiliations:** Department of Nursing Sciences, University of Nigeria Enugu Campus, Enugu, Nigeria

**Keywords:** Theory-practice gap, causes, effects, lived experiences, baccalaureate nursing students, clinical practice, qualitative phenomenology

## Abstract

**Purpose:**

This study evaluated the baccalaureate nursing students’ lived experiences of the causes and effects of TPG during clinical practice in a tertiary health institution in Enugu, Nigeria.

**Method:**

A qualitative design using existential descriptive phenomenological approach was adopted to explore 20 baccalaureate nursing students at 500 level of study. The class level of the students was purposively sampled and the exact number of students sampled using snowball technique. Semi-structured interview guide was the instrument for data collection. Data were collected using audio-tape recorder, face to face in depth discussions, and analyzed using qualitative thematic analysis.

**Result:**

Five broad themes and 12 subthemes emerged from this study namely: Resource constraints (limited resources, resource improvise); Unhealthy human attitudinal and behavioural factors (nurse clinicians, nurse educators, student nurses factors); Environmental system challenges (paradoxical academic design and structure, paradoxical clinical setting); Integration Inadequacy (team cooperation paucity, scarce surveillance, insufficient timing of clinical placement); Observing effects of TPG (observing adverse impacts, observing positive implications).

**Conclusion:**

The causes of TPG during clinical practice emerge from nursing education and practice. TPG have adverse impacts on patients, nursing students, nursing education and practice, other health practitioners, while linkage to response is its positive implication.

## 1. Introduction

### 1.1. Background

Nursing education not only involves theoretical content discussed in classrooms, but also requires sufficient clinical placements to allow for skills development and the application of theoretical content to practice. Unfortunately, the integration of theoretical content in practice does not usually occur smoothly (Monaghem, 2015). From the international perspectives, theory-practice gap (TPG) is viewed as a decade long crisis of competency that continued to plague the nursing community till today (Gamblin, 2019). It has long been highlighted as one of the biggest problems hindering the advancement of nursing sciences and nursing students, and can prevent the baccalaureate nursing students from achieving their learning outcomes (Bouchlaghem & Mansouri, 2018). Salah et al (2018) noted that while the problem of TPG is growing, it is not receiving the attention that reflects its significance, and despite the documentation and researches on it, the improvement on professional practice has been at “snail pace”. Because of TPG, new generations of nurses are being produced unable to cope with the demands of caring (Lindsay, 2019).

Furthermore, Chapman (2017) viewed the TPG in various ways as thus: the differences between idealized practice and common practice, the differences between taught general principles and the difficulty in interpreting them for application to a specific situation, the gap between taught abstract nursing theory and its use to practice and the gap between scientific knowledge and the theory used as common practice. Osuji and El-Hussein (2016) conceptualized TPG as the inconsistencies between what student nurses acquire through theoretical classroom lecture and what is being experienced in the clinical setting. Some of the respondents in the study of Wasini et al. (2019) also declared that the TPG arise when the classroom knowledge is not transferred into practice. Having this in mind, Mahmoud (2014) confirmed the TPG as being a hindrance in student nurses’ clinical learning process.

In addition, Greenway et al. (2019) noted that in Rodger’s Evolutionary Model of concept analysis, antecedents are the causes of TPG which include evidence-based practice, ritualistic practice and education and acquisition of nursing skills. Some researchers argued that the phenomenon of TPG is caused by reasons ranging from certain misconceptions about the exact nature of relationship linking nursing theory and the practice, to specific contributing factors on the part of the students, the nurse educators, the school and the nurse clinicians together with the clinical learning environment (Bouchlaghem & Mansouri, 2018; Dadgaran et al., 2012; Osuji & El-Hussein, 2016). Salifu et al. (2018) identified five themes responsible for TPG in nursing as thus: system inadequacies, resource constraint, challenges of the clinical learning environments, clinical placement and supervision and nurse faculty factors. TPG is also perceived to be created by shortcomings in the following: processes, procedures, education and communication, and occurred when there was lack of evidence-based guidelines and hospital systems were ignored (Jones & Johnstoneb, 2019).

Again, the effects of TPG are the consequences of TPG, which were seen as outcome, portraying what happens after an incidence of concepts occur in Rodgers Model of concept analysis (Foley & Davis, 2017; Greenway et al., 2019). According to the model, TPG was found to consequently influence nurses, nursing students and results to disparity in collaboration between clinical staff and academics. It is acknowledged that TPGs generally and in most cases have negative effects on the learning process and Interfered with evidence uptake in clinical practice (Osuji & El-Hussein, 2016). The discrepancies of TPG have caused much concern among instructors, professors, educators and nursing students (Kermansaravi et al., 2015). Gamblin (2019) stated that the most obvious concern with TPG in nursing is that it endangers patients and places large burden on practices to train their nurses. When gaps were present, patients care was disjointed and lack organization such that normal, expected or planned sequence of events are interrupted, delayed or did not occur as it should (Jones & Johnstoneb, 2019; Salifu et al., 2018). The frustration and difficulties associated with the TPG are largely experienced by nursing students and can have an adverse impact on their socialization into professional role (Al-Awaisi et al., 2015). Also the reality shock introduced by the wide TPG is considered as a major cause of low job satisfaction and high job attrition rates among newly qualified nurses (Al-Awaisi et al., 2015; Monaghem, 2015).

Furthermore, Mohajan (2018) stated that qualitative research methods typically include interviews and observations. In qualitative phenomenological research, lived experience refers to a representation of the experiences and choices of a given person, and the knowledge gained from these experiences and choices (Chandler & Munday, 2011). Phenomenological approach fully describes peoples’ lived experiences of an event by seeking meaning through a detailed exploration of the phenomena through which they live and as described by participants (Thomson et al., 2012). The baccalaureate nursing students are among the major consumers in both theoretical and clinical learning. Mahmoud (2014) confirmed the TPG as being a hindrance in student nurses’ clinical learning process, while Scully (2011, 2022) argued it is the student nurses given their rule-governed state and limited experience who find themselves in the mist of theory-practice void. Therefore the study of their lived experiences of the causes and effects of TPG which exist during the clinical practice is of paramount importance.

Empirically, there is scarcity of primary studies on this topic in Nigeria. The study done in South-east geopolitical zone of Nigeria was on how Clinical Nurse Educators help students come out of TPG using clinical nurse educators as participants (Osuji et al., 2019). The study done using two teaching hospitals in Bayelsa and Enugu states respectively was on knowledge and attitudes of newly qualified staff on theory-practice integration using quantitative approach (Wasini et al., 2019). Also the only study done in Kano State was on knowledge and perceptions of nurses on TPG using quantitative approach too (Abdullahi et al., 2022). Finally, one study done in Ibadan covered what actually constitute the TPG (Odetola et al., 2018).

Meanwhile, some primary studies in developed countries have tried to explore the strategies for bridging TPG in nursing during clinical practice, yet, TPG still exists. Therefore, knowledge of the specific causes of TPG during clinical practice in Nigeria will help the members of nursing profession in avoiding those causes and encourage early preventive measures. Exposing the effects of the TPG will encourage all the stakeholders in nursing profession and policy makers know the adverse impacts of the gap to the individuals involved in receiving and giving care, then intensify efforts to come up with targeted, collaborative and sustainable strategies that can help in bridging the TPG. Globally, this study will also add to existing literatures on the causes and effects of TPG in nursing during clinical practice. The objectives of this study were to: explore the lived experiences of the baccalaureate nursing students on the causes of TPG during clinical practice and examine the students’ observations on the effects of TPG during clinical practice in a tertiary health institution in Enugu.

## 2. Research methods and materials

### 2.1. Research design

A qualitative research design using existential descriptive phenomenological approach by Martin Heidegger (Linsenmayer, 2011) was adopted to allow the investigators describe the current and prevailing issues about the baccalaureate nursing students’ lived experiences of the causes and effects of the TPG during clinical practice. The design was considered appropriate for this study because the nature of the phenomenon of TPG which is filled up with the complexities of subjective human experiences cannot be fully identified using quantitative design. This design was successfully used by Shoghi et al. (2019); Osuji and El-Hussein (2016) and Odetola et al. (2018).

### 2.2. The study site

The study was conducted using 500 level baccalaureate nursing students from the University of Nigeria Enugu Campus (UNEC) that just completed their clinical training experiences at University of Nigeria Teaching Hospital (UNTH) Ituku/Ozalla Enugu in Enugu state of Nigeria. This hospital is the oldest, largest tertiary health institution in the south-east geopolitical zone of Nigeria and the main referral point in Enugu state (University of Nigeria Teaching Hospital Enugu, 2019). It is also the major clinical placement site for the baccalaureate nursing students from the Department of Nursing Sciences UNEC. UNEC is also the sub-campus of the University of Nigeria, Nsukka (UNN), situated in Enugu metropolis. The department was chosen because it is the only nursing department in the state that offers baccalaureate nursing program with Nursing and Midwifery Council of Nigeria accreditation. The department also had an average class size of 161 nursing students and an average students of 800.

### 2.3. Ethics

The researchers obtained ethical approval from the Health Research Ethics Committee (HREC), with approval ethical certificate number UNTH/CSA/329/VOL5/012 of UNTH Ituku/Ozalla Enugu. Further administrative permission was obtained from the Head of Department of Nursing Sciences UNEC. Also the confidentiality of the participants’ information was guaranteed by use of codes like “P1” to “P20” for participant numbers 1 to 20, known only to the participants and the researchers, and used to represent their individual quotes. The settings for the interview was free from public interference. Participants were informed of what the study was about, and freedom to withdraw from the study at any time without any repercussion or prejudice communicated to them. Finally, each of the participants completed a written informed consent form. These were as indicated in the principles of Helsinki Declaration (World Medical Association, 2013, 2022).

### 2.4. Sample/participant recruitment

The participants consisted of 20 volunteer baccalaureate nursing students at 500 level of study. Purposive sampling procedure was used to select the class level of the students. Snowball sampling method was also used to select the exact number of participants who met the inclusion criteria. The first and second participants were recruited through face to face contacts. They gave link to the next three participants that were contacted through phone calls by the first author. The fifth participant gave links to the remaining participants who were recruited through face to face contacts by the first and second authors. This final number was determined after the researchers and the experts (in the use of qualitative design in the Faculty of Health Sciences and Technology, UNEC) agreed that data saturation occurred, and by previous studies on TPG by Saara Kerthu and Nuuyoma (2019) and Odetola et al. (2018).

### 2.5. Instrument for data collections

The instrument used for data collection in this study was the researchers’ designed semi-structured interview guide which was developed by the first and third authors, then reviewed and edited by the second and fourth authors. The instrument has two sections A and B. Section A comprised the participants’ demographic characteristics. Section B contains items designed to address two broad interview subscales (experiences of the baccalaureate nursing students on the causes of TPG and their observations on the effects of TPG during clinical practice), which gave rise to five probing questions (prompts). The probing questions were: Can you describe those factors in your department during classroom teaching and learning experiences which you observed to have contributed to TPG during your clinical practice; Also describe those things you observed as the student-related reasons for TPG; Describe also the reasons you observed from your clinical learning environment which contributed to TPG; From your experiences and observations during the theoretical and clinical training, explain what you noticed about the effects of TPG; How would you describe the positive implications of TPG?

### 2.6. Data collection

Interviews using face to face, with the help of semi-structured interview guide and audio-tape recorder were conducted to assess and extract the experiences and observations of the baccalaureate nursing students on the causes and effects of TPG. Participants who met the inclusion criteria (participants at 500 level of study, have completed the clinical learning experiences, and willingness to participate in the study stated in a written informed consent form) were recruited for the study, contacted individually through telephone and face-to-face. They were asked to provide information on the appropriate day, time and venue for the interview. The face-to-face interview which lasted between 40 to 76 minutes were conducted exclusively in English with each participant separately, going back and forth through the questions until no new information was obtained. The interview format was flexible, conversational, relaxed, and conducted at the most convenient place (departmental garden, hostel and offices in the ward) and time for the participants. The participants revealed their thoughts, observations and experiences regarding the causes and effects of TPG during clinical practice. All conversations were recorded, audio-tapped and field notes written after each shared conversational session, capturing comments, actions and behaviours of individual participants. Information that needed further clarifications were noted and taken back to the participants at the end of each session.

In addition, the researchers in this study also wrote down in a reflexive journal some of their presumptions and thoughts in relation to the phenomenon under study. They also bracketed their thoughts in this topic of existential concerns and made sure they were open to hearing the baccalaureate nursing students’ different experiences and observations concerning the causes and effects of TPG during their clinical practice. Each interview session was terminated when no new idea about the phenomenon emerged from the participants and efforts to continue becomes counterproductive. All the shared conversations were recorded and transcribed verbatim for data analysis. The data collection lasted from June to August 2021, was done by the first and fourth authors, and supervised by the second author.

### 2.7. Data analysis

Data from the individual interviews were analyzed using qualitative thematic analysis to allow for identification of categories, subthemes and broad themes. The analysis was commenced by open and axial coding (Allien, 2017). Initially the transcripts were crosschecked with the audio-tape to ensure correct transcription of the interview. The text-data were read word by word severally to allow for immersion of data in the transcript and to get an overview of the contents. Then, general impression and significant statements relating to the baccalaureate nursing students experiences of the causes and effects of TPG were extracted from each transcript and meanings formulated from them. Responses similar in contents were grouped together (open coding), and this approach was continued until important label for a subtheme emerged. All the data within a subtheme were examined to ensure a fit between data and subtheme. Subthemes were categorized into broad themes (axial coding) by the agreement of the researchers.

Participants’ statements and implicit concepts were reviewed severally, comparing codes and transcripts to ensure subthemes and themes were well developed. Coded subthemes and themes were also reviewed severally to enhance trustworthiness. Investigator triangulation involving the first and second authors, and one independent analyst was done during the data analysis (Bhandari, 2022; Carter et al., 2014). They analyzed the data separately before putting them together. The results of the analysis were discussed by all the four authors, differences and similarities between the authors compared and adjustment made severally until an agreement was reached. The analytical process was also peer reviewed (by a specialist in qualitative nursing research). The findings of this study were then integrated into an exhaustive written description of the fundamental structure of the phenomenon under study.

### 2.8. Trustworthiness

Lincoln and Guba’s parallel criteria (Morrow, 2005) to assess trustworthiness in qualitative research were used in this study, and they include credibility, transferability, conformability and dependability. Credibility (which is the confidence that can be placed in the truth of the research findings) was ensured in this study by prolonged engagement of the participants during data collection, and by peer debriefing. Transferability (how well the findings will be generalized to other settings) was ensured by providing a rich and detailed description of the participants, the setting, variation in participants’ selection (generic and direct entry baccalaureate nursing students), ensuring that data saturation was attained and providing adequate description of the phenomenon (Laumann, 2020; Polit & Beck, 2014). Conformability (ensures objectivity in qualitative research and acts as a check to ensure that the findings are based on the data collected) in this study, was established by using reflexivity. The researchers kept a reflexive jotter (91 pages) throughout the course of the data collection and analysis, which provided evidence of decision process undertaken (Laumann, 2020). Dependability (the stability of the findings over time) was achieved by audit trial (transparent description of the research steps).

## 3. Results

### 3.1. Characteristics of participants

The demographic profile of the participants showed 6 males, 14 females, 5 married, 15 singles, 12 admitted through Joint Admission and Matriculation Board Examination and 8 of them admitted through direct entry. 7 of the participants were within the age bracket of 20-24 years, 11 of them aged 25–29 years old, and 2 were aged 30-34 years. All are Christians. Five broad themes emerged in this study. The summary of the broad themes and subthemes are presented in Table 1.

**Table I.** Summary of the broad themes and subthemes synthesized from the interview (*n* = 20).

Broad Themes	Subthemes
Resource constraints	Limited resources,
	Resource Improvise.
Unhealthy human attitudinal and behavioural factors	Nurse clinicians,
	Student nurses,
	Nurse educators factors
Environmental system challenges	Paradoxical academic design and structure,
	Paradoxical clinical setting
Integration inadequacy	Team cooperation paucity
	Scarce surveillance,
	Insufficient timing of clinical placement
Observing effects of TPG	Observing adverse impacts,
	Observing positive implications

### 3.2. Causes of TPG during clinical practice

#### 3.2.1. Theme 1, Resource constraints

This theme generally describes the baccalaureate nursing students’ experiences of the material and human resource constraints that contributed to TPG during their clinical practice. It led to the development of two subthemes.

##### 3.2.1.1. Limited resources

Participants in this study reported non availability and limited number of human and material resources for the provision of care as among contributory factors to TPG. This limited resources transverse through the academics for students’ learning. The human resources include the nurse clinicians that provide the care and the nurse educators that teach the nursing students. The material resources are in the forms of the equipment and instrument used in the care of the patients, and the ones used in the education of the nursing students. The following quotes illustrates their opinions: “*I was in the ward one day and there was no oxygen to give the patient. No patients’ monitor and sphygmomanometer for vital signs (P2).” “Shortage of staff nurses (P13).” “For examples, bed sheets for bed making are not available most times, materials for bed bathing are never complete and no ward have complete equipment (P12).” “Department of nursing do not have enough lecture halls for the large number of students admitted. There is also shortage of lecturers to cover for the voluminous curriculum (P15).” “There is also lack of adequate practical exam materials leading to poor practical demonstration and learning with resultant poor clinical practice and learning, and this helps in widening the gap (P17)*.

*Non-availability and non-functional equipment e.g., when one want to do wound dressing, he will wait for another to finish dressing wound, soak instrument in “Jik bleach”, and the nurse may not wait patiently for it to soak well before using it for another patient thereby re-infecting wounds*. *We do not have enough dressing forceps, pack trolleys and sterilizer. (P7).*

*No water to work with. Equipment for standard precautions are limited in number e.g., like one wants to wash hand before and after procedure, and there is no water to do that. In fact, I even had to go to neighbouring ward where they have bucket stand for hand washing to wash my hands after carrying out procedure in the ward where I work, and at times, I come to the ward with my own sachet water. Some nurses hoard face masks (P1).*

##### 3.2.1.2. Resource improvise

In this subtheme, the result showed that in order to ensure that procedures are carried out in the clinical area and classroom, improvises were made with available alternatives. *“In removal of stitches, surgical blades are used instead of stitch scissors (P13).” “Improvising in shortage of lecturers, students are used to do the teachings in the form of presentations (P15).”*

*In wound dressing, a lot of improvises are being made like using gloved hand to do wound dressing instead of forceps, use of artery forceps all through in places where dissecting forceps are supposed to be used, use of hands to hold swabs or mopping stick for mopping of trolley (P12).*

*In shortage of staff, at times junior staff nurses are used in managerial responsibilities like auditing where Assistant Directors of Nursing (ADN) and Deputy Directors of Nursing (DDNS) are supposed to be used, and the use of very senior nurses in procedures that junior nurses are supposed to take up (P1).*

#### 3.2.2. Theme 2, Unhealthy human attitudinal and behavioral factors

This theme exposed the reasons emanating from the bad attitudes, behaviours and activities of individual persons involved in giving nursing care and training of the nursing students which caused theory practice gap in nursing during clinical practice. Three subthemes were further generated.

##### 3.2.2.1. Nurse clinicians’ factors

Our data show that nonchalance and aggressive attitudes of some nurse clinicians towards patients, other health care professionals and nursing students affected their work negatively and predisposed to TPG. Statements revealing this include: *“Unhealthy attitudes of some preceptors bring about poor preceptor and student nurses’ relationships and support, and deter students from asking them questions when necessary (P12).”*

*Nonchalance and poor relationship between students and staff, because at times they are not ready to teach us. For example, they address us as “these Bachelor of Nursing Science (BNS) UNEC students” and this affect their willingness to teach and guide us, negatively influences our practical learning and contributes to TPG (P6).*

*Aggressiveness of some staff nurses towards patients, student nurses and other health professionals made us not to learn anything from them, like after one nurse talked to us in an ill-mannered way, she later called us to teach us and we refused to go close to her again (P3).*

*Bad attitudes of some staff nurses, they are not friendly, are not lovable. Some- times when I remember that am going for posting in a particular ward, I get angry and this makes me not concentrate in learning the practical thereby contributing to TPG (20).*

*Some preceptors do not like teaching the baccalaureate nursing students, they pretend not to have time for students. That is the “I do not care attitude.” This leads to poor practical performance for the students which contributes to TPG. They do not give us face mask especially in orthopaedic ward UNTH Ituku, but give to hospital based students from School of Nursing UNTH Enugu (P10).*

##### 3.2.2.2. Student nurses’ factors

In this subtheme, the participants complained that some students skipped classes. Social media activities during classroom teachings and demonstrations, bad friends and going to club distract their attentions, and also affect their clinical practice experiences negatively thereby contributing to TPG. Quotations in this regard include: *“Bad attitudes of some students like skipping of classes, browsing, what-sapping, twitting and face-booking during lectures made students lack concentration which prohibit learning (P20).” “Youthful exuberance, some students go into yahoo, mix up with wrong friends, do drugs and chase women. This shift their attentions and cause some distraction thereby contributing to poor theoretical and practical knowledge and subsequently TPG (P2).” “Lack of personal discipline. They loiter around, thereby not learning what they have come for. Failure to read up procedures before going for practical predisposes to poor knowledge of the practical procedure thereby contributing to TPG (P10).”*

*Poor dedication, late coming, not interested in the duty assigned to her, not willing to do the practice. Not showing interest in the lecture and may fail to do assignment or come to class which could actually make them not to have good practical knowledge. This can lead to widening of the TPG (P3).*

*Some students go for posting to fulfill all righteousness, which make them not to learn well. They keep on pressing their phones when they are on posting in the clinical area which prevent them from learning and this consequently affect their practice and leads to TPG (P19).*

*Nonchalant attitude of the students about their studies and life style (like a student was going to club, bad friends) bring distraction to students’ learning. During the clinical posting, some students will just sign attendance and go. Some of them sit down in the clinical area without observing what some of the senior colleagues are doing, and is affecting clinical practice and contributes to TPG. (P11).*

##### 3.2.2.3. Nurse educators’ factors

Participants also reported on the individual behaviours, attitudes and activities of the lecturers in the Department of Nursing Sciences UNEC that negatively influenced students’ experiences in the clinical area and are linked to TPG. They noted that the hostile nature of some lecturers scare students from asking them questions on areas of the lectures not understood and on issues met in the clinical area. Failure to adhere strictly to the lecture timetable made lecturers extend the lecture hours. These eventually affect clinical practice negatively, contributing to TPG. The following quotes illustrate the participants’ opinions: *“Some lecturers are hostile, we cannot even tell them that we did not understand what they are teaching (P15).” “Students are not able to share their opinion and experiences in the clinical area with the lecturers due to unwelcoming attitudes (P7).” “Poor preparation of lectures and poor teaching methods by some lecturers make them not to lecture effectively (P9).”*

*Once is close to exams, lecturers come and start stressing the students, like 2 hours lecture, the lecturer will spend like 5 hours trying to cover what she failed to cover earlier. Some of them do not adhere to the lecture timetable. The students struggle to pass theoretical exams before thinking of the practical exams. This affects us in the clinical area as we find it difficult to demonstrate the practical skills of what we were taught in the class thereby causing TPG in those aspect of care (P6).*

*Most times some lecturers are not around. During exam period they start rushing lectures, some may not even teach and they give us materials to read*. *The student may not learn that aspect of the theory very well and this eventually affects their clinical practice negatively and leads to TPG (P1).*

*Negligence on the part of some lecturers, they do not follow the timetable. At the end of the day they end up not teaching what they are supposed to teach. The students do not have good knowledge of the practical aspects of the theory, and this is connected to TPG (P20, P2).*

#### 3.2.3. Theme 3, Environmental system challenges

This broad theme represents generally the baccalaureate nursing students’ experiences of the issues and threats from academic and clinical ward environment that caused TPG during the clinical practice. It led to the development of two subthemes.

##### 3.2.3.1. Paradoxical academic design and structure

Participants described the unusual and contradictory academic designs and environment which contributed to the widening of TPG. These were in the forms of voluminous curriculum, poor organization of intensive practical sessions, and inadequate teaching method. Hence the students find it difficult and do not have the adequate knowledge to carry out the nursing care procedures efficiently during clinical practice thereby contributing to TPG. The following statements illustrate the participants’ opinions and experiences: *“Voluminous curriculum; in a nutshell, ‘jack of all trade and master of none’ for the students. Some of the lecturers do not cover the contents of the curriculum and when we get to the ward, we start seeing it (P13).” “Strike and COVID-19 pandemic lockdown extended the curriculum (P2).” “Not organizing intensive practical sessions for us (P6).” “The curriculum is difficult to cover therefore students were not taught all that they are supposed to be taught in the classroom, linking to inadequate clinical learning and practice, then to TPG (P20).”*

*Too many workload for students, they have classes from 8 am to 6pm. All exams are ”jam packed” to last only for one week. So most times things in the ward are strange to the students. They are not learning and practicing well. They prepare for exams just to pass and this contributes to TPG (P3).*

*Poor method of teaching, that is, some of the lecturers use only lecture method Without demonstration. Some students do not learn well with lecture method alone, this negatively affect the practical aspects of those lectures for the student even after graduation, leading to TPG (P9).*

*The lecture method most times, some of the lecturers come to read and go and it affects the students. They do not come with a model to teach and this affects the practice of the procedures that come out of that lecture even as a new graduate. This directly or indirectly contribute to TPG (P7).*

Furthermore, unconducive classroom environment resulting from increased number of students admitted in each academic session was also one of the predisposing factors to TPG experienced by the participants. The classroom is overcrowded and lectures being delivered in unrelaxed condition. *“Increased number of students admitted—students are ‘jam packed’ inside the classroom even with COVID-19 pandemic (P11).” “Unconducive classroom environment because of problem of overpopulation, students stand at the back and outside the classroom* while receiving the lectures. *Both lecturers and students are in a hurry to leave the class (P13).” “The classroom environment is not spacious enough for the population. Students are forced to go out and when they cannot learn in that type of environment, they cannot apply it in the clinical area thereby leading to TPG (P16).” “Too large number of students for a small classroom, recently the number of students admitted are outrageous, making theoretical teaching and learning difficult and this is connected to poor practical knowledge and TPG (P4)” “The classroom is too small for the number of students. Sometimes if there is no light, the classroom is hot, everybody sweat, making learning so difficult (P20).”*

##### 3.2.3.2. Paradoxical clinical setting

This subtheme reflected the participants’ experiences on the problems from the clinical ward environment and structure which contributed in widening of the TPG. These include unconducive practice environment like foul smell and negligence on the part of government in maintaining broken down structure in some wards. Statements revealing this include: *“Structures are broken down and management is not fixing them, therefore the environment is not conducive for the practical procedures, and this worsens the TPG (P4).” “Physical environment like foul smell. For example in Oncology ward, students have phobia for going there and nurses in this ward do the procedures haphazardly to avoid the foul smell. This contributes to TPG (P7).” “Nurses and students do not have enabling environment to practice and this predisposes to TPG (P14).”*

Also, participants observed that the duty rosters are always unstructured and being made in such a way that a few number of nurses cover the shifts. In these situations, there is risk of omitting procedures and not doing some of them well thereby contributing to TPG. Quotations in this regards include:

*Poor structure of duty roster to accommodate less number of nurses to cover the shift and carry out all patient care procedures. This leaves about two nurses covering a whole ward in a shift. Here patients do not receive the necessary care and at times patient relations are left with giving the care. This may expose the patient to danger thereby leading to TPG (P19).*

*The nurses’ duty roster in the clinical area always show a few number of nurses on duty. This makes the workload always much on nurses, they do not have time to carry out all the procedures well or teach the students, and this increases the TPG during clinical practice (P7).*

#### 3.2.4. Theme 4, Integration inadequacy

This broad theme was further disintegrated into two subthemes for better understanding.

##### 3.2.4.1. Team cooperation paucity

In this subtheme, participants reported that there is poor collaboration and communication between the academics and clinical area. There is also no team work between the staff nurses and students, other health care team members, hence, no shared interest and solution to any existing problem. All these are linked to TPG during clinical practice. The following quotes describe this subtheme: *“No teamwork between staff and students. Poor collaboration and communication between lecturers in academics and nurse clinicians (P12).” “There is problem between doctors and nurses at UNTH Ituku/Ozalla, conflicts of ideas and no communication between them. Divergence collaboration between academics and clinical area. Issues relating to theoretical and clinical trainings are not shared between them. This affects the work and causes TPG (P15).” “Poor communication between students and staff and discrimination from doctors. This discourages students and brings much gap (P7)” “No teamwork between staff and students, also between academics and clinical area. Both groups appear to be too busy to make out time to discuss with each other thereby contributing to TPG (P1).” “Poor inter and intra professional, and interpersonal work cooperation to discuss on the problem areas during clinical practice widen the TPG that already exist (P16, P13).”*

*In classroom, they teach team work between health care personnel, but in practice, doctors impose superiority on nurses and lab scientists trying to show their relevance, and patients tend to suffer it, because “when two elephants fight, the grass suffers,” This contributes to gap (P5).*

##### 3.2.4.2. Scarce surveillance

The result of this study showed that inadequate supervision and auditing of nursing care procedures and lectures contribute to TPG during clinical practice. The participants felt that there is poor supervision of student nurses by the staff nurses, clinical instructors and preceptors in the clinical area when the students are carrying out procedure. In this situation, the students’ errors are not detected and corrected or guided during those procedures and this contributes to TPG. This was manifested in statements like: *“Poor and uncoordinated supervision of student nurses by the staff nurses, clinical instructors and preceptors, like in the dressing of wound, no body come to inspect me (P20).” “Clinical instructors do not often come for supervision in the clinical area. Eventually, when they come, they are concerned with the attendance, no attention on the procedures (P4).” “No supervision of student nurses, some nurses just assign duties to us and go, they do not know when things go wrong in the procedures and this contribute in widening the gap in those procedures (P15).”*

Secondly, it was reported that poor delegation of duties and auditing of the activities of nurse clinicians in discharging their duties bring about TPG. Through this, the errors and haphazard ways of carrying out procedures by some nurses are not observed and corrected. The participants also felt that poor supervision and auditing of the activities of the lecturers during classroom teaching and practical demonstrations contribute to TPG. *“Lack of impromptu auditing of the lecturers during the lectures in the classroom and practical demonstration makes them to continue with the same errors which directly or indirectly affect the students’ clinical performances negatively, thereby predisposing to TPG (P19).” “Poor auditing of the students’ results from which lecturers’ capabilities are judged bring about TPG during clinical practice. This is because corrections are not enforced on those lecturers to implement positive change on their lecture methods (P6)”*

*Some senior nurses assign duties to junior ones and not supervise it. There is poor auditing of the activities of the nurses and poor supervision of procedure. In theory, they talked about bureaucracy and hierarchy in the ward, it is not so. For example, I witnessed a Principal Nursing Officer shout at ADN because the ADN is not assertive in supervision (P5).*

*Poor monitoring and auditing of what and how some of the lecturers teach. This do not encourage positive change in the areas the teachers have gone wrong thereby giving the students inadequate knowledge which is carried to the clinical area, thereby contributing to TPG (P10).*

##### 3.2.4.3. Insufficient timing of clinical placement

A few participants in this study reported that short semester and short time of clinical posting result to short duration of clinical posting and inadequate coverage of posting to speciality areas. Procedures arising from those speciality areas may not be well practiced thereby contributing to the widening of the gap in those clinical procedures not adequately covered. This was expressed in quotations like: *“Poor structure of the clinical posting time table results to inadequate coverage of the clinical posting experiences, leading to poor practice of procedures with resultant TPG (P15).” “Poor time management and short time of posting bring about poor coverage of practical procedures thereby causing inadequate knowledge of those procedures not covered and this can cause TPG (P12).” “Short semester resulting to inadequate time to cover for all the specialist clinical sections during clinical posting contribute to TPG (P14).”*

### 3.3. Effects of TPG

#### 3.3.1. Theme 5, Observing effects of TPG

This theme describes generally what the participants observed as the effects of TPG during clinical practice. Two subthemes were extracted from this broad theme.

##### 3.3.1.1. Observing adverse impacts

Participants’ descriptions showed that TPG have bad effects on the patients, nursing students, nursing education, nursing practice and other health care professionals. According to them, TPG affects the patients physically, psychologically and economically. *“TPG leads to complications like wound contamination and breakdown, delay recovery, long hospital stay, poor satisfaction with the care received and disability (P15).” “With TPG, patients do not receive quality care because the care is ‘half baked’. There is increased mortality and morbidity in patients (P17).” “With TPG, patients spend more because they stay longer than they should in the ward (P18).”*

*Patient suffer it a lot. For example, a situation in one of the wards at UNTH Ituku/Ozalla, where a patient was coughing out blood, the doctors came and wrote drugs that should be administered and the student nurse informed the staff nurse in nurses’ office. The nurse told the student to go and write “patient refused to take the drug” in the patient’s folder. But the student did not have the conscience to do that and the patient eventually died. “Avoidable death” (P6).*

*The patients come down with nosocomial infection, prolonged hospital stay leading to high cost of care. Give patients bad impression about nurses. They start failing their appointments, prefer not to come to UNTH Ituku/Ozalla and rather go to private hospital, with the worst effect being complications resulting to death (P1).*

*With TPG, patients do not recover well, they recover with complications. For example, some patients go to theatre for surgery up to five times and their problems are not fixed. This can cause adverse damage to patients’ condition and the error can even lead to death (P4).*

Secondly, the participants reflected their experiences and observations of the negative effects of TPG on the nursing students as thus: *“TPG reduces our self-confidence and make us have poor delivery of care (P8).” “Reduce clinical skills of students due to the TPG, raised anxiety during clinical exam and reduced our level of assertiveness (P14).” “Poor quality of practical knowledge, incompetency, frustration for students and longer stay in school (P18).” “Poor clinical experiences of the nursing students thereby not learning on patients’ care, doing the work haphazardly and reduces the quality of care being rendered to patients (P20).”*

*Confusion, like which one to move with? They taught us this in the class and we are seeing another thing in the clinical area. Wrong knowledge, we come to the ward practicing wrong thing. It becomes part of one to transfer wrong knowledge, and the students may not know it (P1).*

In addition, the baccalaureate nursing students also shared their experiences and observations on the negative effects of TPG on nursing education as follow: *“Production of non-proficient and incompetent students and nurses (P1).” “Leads to limitation in the number of nurses who major in nursing education and nurses will not see the need to specialize in nursing education (P16).” “TPG brings more workload to nurse educators because there are areas that will be added to the curriculum for them to cover, making it more cumbersome (P6).” “Poor growth of nursing education (P12).” “TPG reduces the standard of nursing education (P2).” “Nurse Educators are demoralized to teach, and the students still exposed to the TPG in the hospital, with the worst effect being embarrassment of student nurses (P18, P13).”*

*TPG in nursing during clinical practice affects nursing education negatively by reducing the interaction in the class between the lecturers and the students because the theoretical knowledge is not well matched with the practice, and the students do not relate well with some of the lecturer to discuss the problem and some concepts are also abstract (P14).*

Poor projection of the good image of the profession to the outside world, poor progress of the profession and non-productive nurses were also identified as the negative impacts of TPG to nursing profession. Statements revealing these include: *“TPG gives bad name to nursing profession because people see it that we are not competent to discharge our duties (P16).” “Patients and others see nurses as those that do not know what they are doing, and thereby making them look like ‘quack’ nurses. (P20).” “The nursing profession gradually loses its relevance because of incompetency (P10).” “It seems as if members of nursing profession are not practicing what they are supposed to practice. Nurses may not make good recommendation for relatives to enter the profession because of job dissatisfaction (P9).”*

Furthermore, participants described the negative effects of TPG on other health care professionals like doctors, lab scientists, nutritionists, physiotherapist and the hospital management. *“TPG in nursing have caused some decreased inter professional collaboration because other health professionals think we do not have anything to offer leading to conflicts (P17).” “With TPG in nursing during clinical practice, the hospital is blamed for poor patient care. (P7).” “TPG in nursing affects other health care professionals negatively. They make use of nurses’ documentation in their care and if nursing care are not documented, patients’ care are wrongly planned (P20).” “TPG in nursing during clinical practice leads to a kind of confusion in the duty of other health care professionals because they begin to ask so many questions about the nursing care (P4, P10).”*

*Doctors do not get accurate information about the patients and this can lead to poor medical information. Even the physiotherapist, medical lab scientist and nutritionist are affected because they do not get accurate information about the patients due to the gap. These other health care professionals have used and can still use the gaps in nursing procedures to do litigation against nurses (P12).*

##### 3.3.1.2. Observing positive implications

In this subtheme, some of the participants did not see and believe that there are positive implications of TPG in nursing. Quotations revealing this include: *“There is nothing positive I feel about TPG in nursing (P10).” “It is not a good thing, therefore, it does not have a positive implication (P2).” “Most of the times, it gets worse (P4).” “I do not think TPG in nursing during clinical practice have any positive implication (P9, P12, P19 and P20).”*

In contrast to the above statement, some of the participants described the positive implication of TPG as having linkage to response and solution. These quotes represent their individual views: *“If TPG in nursing during clinical practice goes on for a long time, it attracts the attention of the policy makers (P3).” “TPG will encourage the lecturers impact the knowledge and the students to work harder to learn better (P15).” “I do not think there is any positive implication, except if the nurses, educators, student nurses and management review the gap and work on it for solution (P12).” “Presence of gap may equip the nurses to know how to improvise and manage in emergency situations (P5).” “TPG helps the clinical nurses to plan and institute appropriate interventions (P14).”*

## 4. Discussion

The limited material resources noted to have contributed to TPG in this study is thought to have caused delayed care, prevented provision of right care, got nursing students confused as some of the procedures were either not done or done the wrong way different from what was taught in the class theoretically. Hence, there is tendency of some students and nurse clinicians to record assumed values. This is blamed on the delay in making and following up requests for the provision and repair of those materials by nurse administrators, poor inventory of the available ones, delay in the procurement and repair of those materials by the hospital and university management. The limited human resources might result from poor salary, general hardship and insecurity which led to mass exodus of nurses to developed countries where they are better valued, paid and with good working environment. It was confirmed in the studies of Salifu et al. (2018); and Wasini et al. (2019) that the general lack of resources is a main contributor to TPG. Improvising material resources made the nurse clinicians to render care and services using equipment that are not well designed for the procedure, got used to wrong steps of carrying out procedures and see those wrong steps as normal routine. Nursing students were not able to experience the correct standard procedure being carried out using ideal instrument. The students used in place of lecturers might not have explained the points very well, and this is linked to poor performance during clinical practice thereby causing TPG. Salifu et al. (2018) found out in their study that clinicians often adopted unconventional (getting used to it and improvise) approaches to clinical procedures and patient care activities which were not in consonance with textbook orders.

Unfriendly and nonchalant attitudes of some nurse clinicians identified as predisposing factor to TPG in this study, might have resulted to omissions of important steps in nursing care procedures and put patients’ lives at risk. It severe the relationships and support within the nurses, between the nurses and other health care professionals, scare the students away from the staff nurses and deter their practical learning, leading to errors and subsequent TPG. The study of Saara Kerthu and Nuuyoma (2019), showed that the discriminatory attitudes of the health care providers towards students in clinical settings resulted to gap. The non-teaching and support of the students by the preceptors identified in this study might be due to fear of future seniority, poor motivation of nurses and much workload on a few nurses on duty. It might also be because of dual roles and absence of preceptor training. They might also not be paid additional incentives for that role, thereby contributing to the relapse in their roles as preceptors. Their duties as nurse clinicians in the ward also suffer as some of the procedures are omitted in the process or not done well. The complexity of these roles in a short staffed clinical environment contribute to TPG. This is in agreement with Salifu et al. (2018) who noted that the preceptor system was ineffective because of lack of preceptor training together with multiple roles they assume. This result also concurred with that of the findings of Salifu et al. (2018) which showed rivalry and power play among university undergraduate nursing students, hospital-based student nurses and staff nurses.

Bad behaviours of students identified to have linked to TPG during clinical practice in this study might be associated with poor orientation of these students on the implications of such act and the far distance of the university from the clinical area. The above result aligns with that of the study of Botma & Mackenzie (Botman & Mackenzie, 2016) where nurse clinicians and clinical preceptors perceived students as uninterested, disrespectful and with preferences of whom they want to work with. Non-strict adherence to lecture timetable and poor teaching method by some nurse educators are blamed on shortage of lecturers, poor monitoring and auditing of lectures, poor sponsorship and non-attendance of workshops on innovative practices in nursing education. The hostile nature of some lecturers is also thought to arise from the personality dispositions of those lecturers and ill-mannered attitudes of some of the students. This result corroborates with the suggestion in the study of Salifu et al. (2018) that most nurse faculties were either inexperienced or had lost touch with the realities of nursing practice due to prolonged absence from active clinical work.

In paradoxical academic design and structure, the poor coverage of the curriculum and non-organization of intensive practical in the practicum are blamed on the mass attrition of nurse educators to developed countries, recurrent industrial actions by the Academic Staff Union of the University (ASUU) and COVID-19 pandemic lockdown. Also the admission of excess number of nursing students by the department compared to the size of the classroom might result from political interferences in students’ admission which left the department with no control on students’ admission. The result of this very study is in line with that of Salifu et al. (2018) which found out that the educational designs and the implementation of nursing education curriculum did not promote practical skills acquisition and preparation of nursing students to face the realities of clinical nursing practice. They also noted that challenges from the numbers of students admitted is another fundamental issues in nursing education that contributes to TPG. The unconducive practice environment described as having linkage to TPG, might be connected to lack of commitment by hospital management to repair broken down structures. The poor structure of the nurses’ duty roster brought about having a few number of nurses on duty, hence, the risk of not carrying out all the nursing care procedures, thereby leading to TPG. Feti (2019) reported that clinical environment characterized by poor hospital condition contributed to TPG.

In team collaboration inadequacy, the poor communication and team work between the academics and the clinical area and between nurse clinicians and nursing students might be attributed to intra-professional rivalry and heavy workload on both sides. They appear to be too busy to communicate with each other, and there is no enforcement for such communication by the nurse administrators and the academics. With this, issues that move the profession forward are not communicated and shared. Also, because of inter-professional conflicts and rivalry, important issues concerning patients’ care are not freely shared with resultant omissions and errors in care. All these widen the TPG. Safazadeh et al. (2018) stated that in organizational relationship, shortage of communication, poor teamwork between the nurses and other health care professionals and mistreatment of supervisors towards students were the reasons for TPG.

The poor supervision and auditing of the activities and work of student nurses, nurse clinicians and lecturers by the clinical instructors, nurse administrators, and senior nurse educators respectively might be connected to heavy workload of combining their routine primary ward assignments with the supervisory and auditing roles. The far distance of the clinical area from Enugu metropolis where most of nurses reside might have also deterred the clinical instructors from exercising their supervisory role regularly. Again, absence of strict laid down policies and procedures on supervision and auditing bring about role conflict on this. All these negatively influence nursing care procedures and are connected to TPG. This result echoed that of Safazadeh et al. (2018) that scarce supervision of nurses’ work, and part-time presence of clinical instructors are some of the reasons for TPG.

Minimal satisfaction with the care received, delay recovery, raised economic burden on patients and relations, and increase morbidity and mortality might be attributed to the facts that struggles to reduce the TPG did not work out. Some of these patients in the hospital come on referral bases, might also not be literate enough to be aware of the existence of “patients” bill of right”, and are not likely to take measures against the poor nursing care services received. Massey et al. (2017) stated that TPG in nursing practice leads to worsening of patient’s condition, delay in patients’ recovery, and death.

Poor knowledge and incompetence in carrying out procedures among the nursing students resulted to some of the students dodging those procedures. This exposes them to poor proficiencies in those practical procedures where TPG exist. The students might not be able to express their views in discussions related to those area they are deficient in during clinical practice. Greenway et al. (2019) found out in their study that TPG causes barriers to skill acquisition and feeling of incompetency.

As some of the nurse clinicians and nursing students become non proficient in some clinical skills, it gives the interpretation that those aspects of the procedures were not well taught and that the curriculum on that were not well covered during the training. Therefore, this gives wrong impression about the activities of nurse educators in the academics and might deter the students and nurses in advanced nursing studies from specializing in nursing education, with resultant poor growth of nursing education. The more workload and cumbersome curriculum on nursing education might place a large burden on practice to train their nurses (Gamblin, 2019). If there is no adequate theory practice integration in relation to the curriculum implementation, nursing education may not be able to change the life of respective society (Salifu et al., 2018).

Reduced level of assertiveness among members of profession and setback in the professional practice of nurses identified in this study might be connected to incompetency among some nurse practitioners, poor involvement in research, and practices not based on research evidence. All these culminate to poor growth of the profession and non-productive nurses identified in this study. Bouchlaghem and Mansouri (2018) in their result noted that incompatibility of theoretical education with the performance of nurses in the clinical setting can lead to inappropriate use of scientific evidence, resulting to the decline in the quality of nursing services.

Decreased inter professional collaboration, litigations and conflicts revealed in this study might be related to the fact that doctors and other health care professionals rely on the documentations by nurses during patients’ care. So when there are errors or delay in nursing care, confusion arises leading to wrong medical diagnoses and treatments. The nursing care services represent reasonable percentages of the total services patients receive in the hospital, hence the blame on the management for TPG in nursing. Greenway et al. (2019) shared similar opinion that TPG brings about disparity in collaboration between clinical staff and academics which is manifested by incomplete, and sometimes meaningless interactions within and between the clinical environment, other health care professionals and the school.

The aspect of TPG not having any positive implication in this study might be suggestive of the fact that efforts by a few nursing stakeholders are minimal, and that the government is not giving maximum attention to health care sector. The result of the TPG in nursing having linkage to the response and solution in this study might be connected to the fact that continuous TPG during clinical practice might stimulate future researches by the policy makers and stakeholders in nursing profession on the targeted and sustainable strategies to handle it. This result corroborates with the findings of the study of Osuji and El-Hussein (2016) where a few participants viewed TPG as a potential positive stimulus to the students process of learning. It also concurred with that of the qualitative study of Ferraz et al. (2020) which suggested that with TPG, new knowledge was generated through multiple and repeated lessons.

## 5. Conclusion

The causes of TPG during clinical practice emerge from nursing education and practice. TPG have negative impacts on the patients, student nurses, nursing practice, nursing education and other health care practitioners, while linkage to response is its positive implication. Therefore, strategies to bridge the TPG should be targeted to the specific causes and effects.

## 6. Implication of the study for nursing practice

There is need for members of nursing profession to recognize and have adequate understanding of these specific causes of TPG, develop positive attitudes, and work towards avoiding them, then advice the university, hospital management and policy makers on them. It is pertinent that the nursing stakeholders evaluate the identified adverse effects of TPG, draw a work-plan for targeted, collaborative and sustainable strategies for bridging the TPG. The absence of positive implications of TPG signifies that the effects are worsening, while the revelation of linkage to response signifies hope for improvements. It also avails the nurse administrators the opportunity of consistently reminding the policy makers of the importance of implementing and monitoring those strategies for closing the gap, thereby relieving the burden of the TPG on the vulnerable groups.

## 7. Limitation of the study

This study had few limitations. The range of experiences on this study was limited as no 400 level baccalaureate nursing was interviewed. The attempt to interview them proved abortive as they did not meet the inclusion criteria (having not had adequate clinical experiences). Though the authors took several steps to ensure trustworthiness, the generalizability of the findings of this study was limited by the choice of the design (qualitative). This is because the purpose of qualitative research is directed towards providing in-depth explanation of the meaning of the phenomenon as lived and experienced by each participant, rather than generalizing findings. Secondly, analytical generalizability in qualitative studies is meant to generalize from particularities to theories (Polit & Beck, 2010; Smith, 2017). It was also shown in the study of Carminati et al. (2018) that generalizability is possible in qualitative study provided that it is the main objective of the study. The participants were told to maintain confidentiality of the things discussed during individual interview, however the researchers were not sure that was done. There is also limitation of literatures on this topic in Nigeria.

### 7.1. Geo-location information

UNEC is located inside Enugu town behind Independent Layout. Its address is College Road Ogui New Layout in Enugu North Local Government Area of Enugu State, Nigeria. It is surrounded by Kenyetta market to the west, Independent Layout to the north and Ogui New Layout to the east and south. The zip code for UNEC is 4,000,241, while the website is www.unn.edu.ng. Its coordinate is 6° 25’ 30. 7” N7° 30 - - -. The latitude is 6. 45381, and the longitude is 7.5277. The Department of Nursing Sciences is within the Faculty of Health Sciences and Technology, located at the north-east of the university from the entrance gate.
